# The persistent risk of secondary malignancies in gastric neuroendocrine tumor survivors: a population-based analysis

**DOI:** 10.1007/s10238-025-01706-y

**Published:** 2025-05-14

**Authors:** Yuheng Ding, Jun Liu, Lingna Shen, Zhipeng Yan, Yonghong Huang, Yihui Huang, Rong Huang, Yunda Qian, Xiaojun Lou, Lai Wang

**Affiliations:** 1 Department of Gastroenterology, Jiaxing Hospital of Traditional Chinese Medicine, Jiaxing, 314000 Zhejiang China; 2 Department of Internal Medicine, Jiaxing University Affiliated TCM Hospital, Jiaxing, 314000 Zhejiang China; 3 Hangzhou Institute of Medicine (HIM), Zhejiang Cancer Hospital, Chinese Academy of Sciences, Hangzhou, 310022 Zhejiang China

**Keywords:** Gastric neuroendocrine tumors, Second primary malignancy, Risk factors, Standardized incidence ratios

## Abstract

Gastric neuroendocrine tumors (G-NETs) are rare neoplasms with a favorable survival rate, yet they present a significant risk for second primary malignancies (SPMs). This study aims to estimate the relative risks of SPMs in G-NET survivors, exploring variations across key patient characteristics. Patients diagnosed with G-NETs were identified from the Surveillance, Epidemiology, and End Results database (2000–2021). Standardized incidence ratios (SIRs) and excess absolute risks (EARs) were calculated to assess SPM risk stratified by age at diagnosis, gender, race, latency period, marital status, and surgical intervention. Among 5072 G-NET survivors, 912 (18.0%) developed SPMs, with a median interval of 34.3 months between the diagnoses. The overall SIR for SPMs was 2.09 (95% confidence interval [CI] 1.96–2.23), corresponding to an EAR of 145.64 per 10,000 person-years. Increased risks were observed for cancers of the stomach, small intestine, thyroid, hepatobiliary system, pancreas, and esophagus. The highest risk for SPMs occurred within the first 4 years following G-NET diagnosis (SIR 2.57; 95% CI 2.11–3.1), with a gradual decline thereafter. Patients under 50 years had the highest SIRs, particularly for stomach cancer (SIR 196.28; 95% CI 160.21–238.05). Females exhibited a slightly higher SIR than males. White patients demonstrated the highest risk for stomach cancer, with an SIR of 63.88 (95% CI 57.00–71.38). G-NET survivors are at a persistently elevated risk of developing SPMs, particularly within the first 4 years of diagnosis. Age, gender, and racial factors significantly influence this risk. Personalized surveillance strategies are warranted to address these disparities and reduce SPM incidence.

**Supplementary Information:**

The online version contains supplementary material available at 10.1007/s10238-025-01706-y.

## Introduction

Gastric neuroendocrine tumors (G-NETs) are a rare and distinct category of neoplasms that originate from the enterochromaffin-like cells within the gastric mucosa [1–3]. In recent decades, advances in early detection and treatment strategies have significantly improved the survival rates for individuals diagnosed with G-NETs [4]. The evolving systemic treatment scenario for G-NETs encompasses significant advancements, including the use of somatostatin analogs (SSAs) for hormone control, peptide receptor radionuclide therapy (PRRT) for metastatic disease, and targeted therapies such as tyrosine kinase inhibitors (TKIs) and mTOR inhibitors. Additionally, emerging evidence suggests that immune checkpoint inhibitors (ICIs) and novel chemotherapy combinations may offer therapeutic benefits, particularly for high-grade or poorly differentiated G-NETs [5–9]. However, the long-term health outcomes for these survivors remain an important area of investigation. A critical concern is the heightened risk of developing second primary malignancies (SPMs), which poses an ongoing challenge for clinicians and researchers [10–13].

The clinical characteristics and risk factors associated with SPMs in G-NET survivors warrant dedicated research. While existing studies often treat G-NETs as part of the broader category of neuroendocrine tumors (NETs) due to their shared origin from neuroendocrine cells and similar pathological features [14–16], this approach may obscure the unique risk profile of G-NET survivors. As a result, these studies may misrepresent the specific risk factors associated with SPMs in G-NET survivors, potentially leading to generalized surveillance protocols that fail to address the distinct needs of this population. Specific demographic and lifestyle factors impacting G-NETs survivors may differ, highlighting the need for more targeted research in this area.

This study seeks to assess the relative risks of all types of SPMs and specific malignancies in G-NETs survivors. Additionally, it aims to examine how these risks differ according to key patient characteristics, including age at diagnosis, gender, race, latency period, marital status, and type of surgical intervention. By identifying distinct risk factors, this research seeks to inform more tailored and effective screening strategies for G-NET survivors, ensuring that their health outcomes are managed with precision and individualized care, rather than relying on generalized protocols for other NET subtypes.

## Material and methods

### Data source

The dataset for G-NETs was sourced from the Surveillance, Epidemiology, and End Results (SEER) database, November 2023 Submission (2000–2021), encompassing 17 registries (excluding Alaska) and representing approximately 26.5% of the U.S. population (http://seer.cancer.gov). Detailed demographic and clinical data, including age at diagnosis, gender, race, marital status, sequence number, cancer stage, and treatment information, were retrieved using the Multiple Primary Standardized Incidence Ratio (MP-SIR) and Case Listing Sessions of the SEER *Stat software, version 8.4.4. As the SEER database is publicly accessible and anonymized, the requirement for ethical approval was waived.

### Data collection

Patients diagnosed with a first primary G-NET between 2000 and 2021 were included in this study. Tumors were limited to the stomach (C16.0–C16.9) according to the classification criteria outlined in the Third Edition of the *International Classification of Diseases for Oncology* (ICD-O-3). Eligible cases were identified based on a "Yes" designation for the first primary malignancy indicator and a behavior code indicating malignancy. To ensure the cohort specifically represented neuroendocrine tumor, only patients with ICD-O-3 tissue/behavior codes of 8240/3, 8241/3, 8242/3, 8243/3, 8244/3, 8245/3, or 8249/3 were included. The following exclusion criteria were applied: (1) a latency period of fewer than 2 months between the initial primary malignancy and SPM to exclude synchronous tumors. Specifically, if a second malignancy was diagnosed within 2 months of the initial G-NET diagnosis, it was not considered an SPM. This approach is consistent with methodologies used in previous studies analyzing second primary malignancies, as it helps to establish a minimum interval between diagnoses, reducing the likelihood of including tumors that may have co-occurred or were already present at the time of the initial diagnosis [17]; (2) incomplete or ambiguous information regarding follow-up duration or latency intervals; and (3) cases diagnosed solely via autopsy or death certificates. SPMs were defined as subsequent malignancies, identified using clinical documentation specifying "malignant tumors for patient" and the sequence number associated with multiple primary malignancies.

### Statistical analysis

Descriptive characteristics across various groups were analyzed using the Chi-Square (x^2^) test for categorical variables and Analysis of Variance (ANOVA) for continuous variables. The risk of SPMs in patients diagnosed with G-NETs was analyzed by calculating standardized incidence ratios (SIRs) and excess absolute risks (EARs) per 10,000 person-years. The SIR was calculated as the ratio of observed SPMs in our G-NET cohort to expected SPMs based on age-, sex-, race-, and year-matched cancer rates in the general U.S. population. Expected counts were derived using SEER*Stat software, which accounts for person-years at risk and applies population incidence rates to our cohort. Confidence intervals (95% CI) were estimated using standard statistical methods for rare events. EAR represents additional SPM cases per 10,000 person-years compared to the general population. To provide a more comprehensive understanding, the analysis was further stratified based on demographic and clinical variables, including age at initial cancer diagnosis, sex, race, latency period, marital status at initial cancer diagnosis and surgery.

## Results

### Characteristics of patients

Between 2000 and 2021, a total of 5,072 individuals diagnosed with histologically confirmed G-NETs were identified from the SEER-17 database. Demographic analysis revealed that the majority of patients were female (3302; 65.1%), with racial distribution showing 77.6% (3,936) identifying as White, 13.4% (678) as Black, and 9.0% (458) as belonging to other racial groups. The largest proportion of diagnoses occurred in individuals aged 50–69 years (2633; 51.9%), and 62.8% (3187) of the cohort received surgical treatment for their initial G-NETs. During the observation period, SPMs were diagnosed in 912 patients (18.0%), with a median interval of 34.3 months between the first and second cancer diagnoses (Table 1). The mean age at the first G-NET diagnosis was 60.3 years, and the median follow-up duration was 79 months.

**Table 1:** Patient demographics of the study cohort, Surveillance, Epidemiology, and End Results 2000–2021

	All patients (*n* = 5072)	None SPM (*n* = 4160)	With SPM (*n* = 912)	*P*
Age at diagnosis of first malignancy				< 0.01
< 50 years	1089 (21.5%)	915 (22.0%)	174 (19.1%)	
50–69 years	2633 (51.9%)	2112 (50.8%)	521 (57.1%)	
≥ 70 years	1350 (26.6%)	1133 (27.2%)	217 (23.8%)	
Gender				< 0.01
Male	1770 (34.9%)	1416 (34.0%)	354 (38.8%)	
Female	3302 (65.1%)	2744 (66.0%)	558 (61.2%)	
Race				0.03
White	3936 (77.6%)	3200 (76.9%)	736 (80.7%)	
Black	678 (13.4%)	567 (13.6%)	111 (12.2%)	
Others	458 (9.0%)	393 (9.4%)	65 (7.1%)	
Marital status at diagnosis of first malignancy				< 0.01
Married	2666 (52.6%)	2142 (51.5%)	524 (57.5%)	
Unmarried	2406 (47.4%)	2018 (48.5%)	388 (42.5%)	
Year of diagnosis of first malignancy				< 0.01
2000–2010	1780 (35.1%)	1391 (33.4%)	389 (42.7%)	
2011–2021	3292 (64.9%)	2769 (66.6%)	523 (57.3%)	
Tumor stage of first malignancy				< 0.01
Localized	3765 (74.2%)	3088 (74.2%)	677 (74.2%)	
Regional	182 (3.6%)	168 (4.0%)	14 (1.5%)	
Distant	232 (4.6%)	211 (5.1%)	21 (2.3%)	
Unstaged	893 (17.6%)	693 (16.7%)	200 (21.9%)	
Received surgery for first malignancy				0.75
Yes	3187 (62.8%)	2622 (63.0%)	565 (62.0%)	
No/unknown	1885 (37.2%)	1538 (37.0%)	347 (38.0%)	

SPM, second primary malignancy

#### Risk and burden of SPMs

**Fig. 1:**
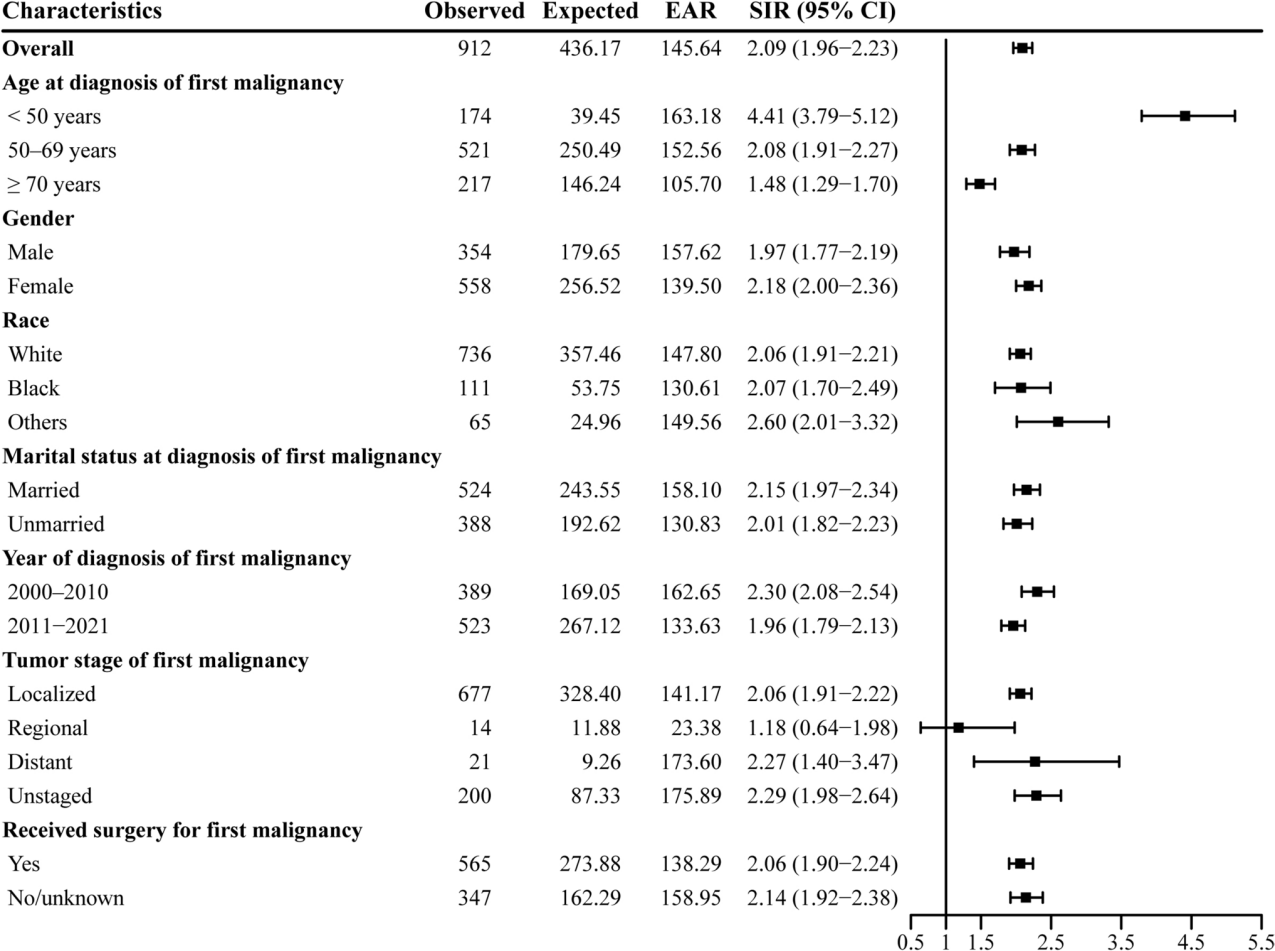
Risk of second primary malignancies according to characteristics of gastric neuroendocrine tumors, Surveillance, Epidemiology, and End Results 2000–2021. CI, confidence interval; EAR, excess absolute risk; SIR, standardized incidence ratio

The overall risk of developing an SPM following the first G-NET diagnosis was significantly elevated, with an SIR of 2.09 (95% CI 1.96–2.23) and an EAR of 145.64 per 10,000 person-years (Fig. 1). Solid tumors represented the majority of these SPMs, with an SIR of 2.24 (95% CI 2.09–2.40). Notably, increased risks were observed for cancers of the stomach (SIR 56.67; 95% CI 51.13–62.65), small intestine (SIR 8.12; 95% CI 4.89–12.69), thyroid (SIR, 3.34; 95% CI 2.18–4.89), hepatobiliary system (SIR 3.33; 95% CI 2.36–4.57), pancreas (SIR 2.84; 95% CI 2.03–3.87), and esophagus (SIR 2.50; 95% CI 1.20–4.60) (Table 2).

**Table 2:** Risk of specific second primary malignancies after first primary gastric neuroendocrine tumors by sex in the USA from 2000 through 2021

	All				Male				Female			
	O	E	SIR (95% CI)	EAR	O	E	SIR (95% CI)	EAR	O	E	SIR (95% CI)	EAR
All sites	912	436.17	2.09 (1.96–2.23)	145.64	354	179.65	1.97 (1.77–2.19)	157.62	558	256.52	2.18 (2.00–2.36)	139.5
All solid tumors	848	378.65	2.24 (2.09–2.40)	143.65	326	154.81	2.11 (1.88–2.35)	154.76	522	223.84	2.33 (2.14–2.54)	137.97
Oral cavity and pharynx	14	9.71	1.44 (0.79–2.42)	1.31	12	5.71	2.10 (1.09–3.67)	5.68	2	3.99	0.50 (0.06–1.81)	− 0.92
Esophagus	10	4	2.50 (1.20–4.60)	1.84	5	2.71	1.80 (0.60–4.31)	2.07	5	1.29	3.88 (1.26–9.05)	1.72
Stomach	382	6.74	56.67 (51.13–62.65)	114.86	115	3.32	34.65 (28.61–41.60)	100.96	267	3.42	78.02 (68.94–87.96)	121.97
Small intestine	19	2.34	8.12 (4.89–12.69)	5.1	13	0.94	13.77 (7.33–23.54)	10.9	6	1.39	4.30 (1.58–9.37)	2.13
Colorectal	32	38.18	0.84 (0.57–1.18)	− 1.89	15	15.78	0.90 (0.53–1.57)	− 0.7	17	24.32	0.70 (0.41–1.12)	− 3.39
Hepatobiliary	38	11.42	3.33 (2.36–4.57)	8.14	22	5.91	3.72 (2.33–5.64)	14.55	16	5.51	2.90 (1.66–4.72)	4.85
Pancreas	40	14.08	2.84 (2.03–3.87)	7.93	11	5.43	2.02 (1.01–3.62)	5.03	29	8.64	3.35 (2.25–4.82)	9.42
Lung and bronchus	63	59.44	1.00 (0.81–1.36)	1.09	25	23.97	1.04 (0.67–1.54)	0.93	38	35.46	1.07 (0.76–1.47)	1.17
Melanoma of the skin	13	20	0.60 (0.35–1.11)	− 2.14	9	10.26	0.88 (0.40–1.67)	− 1.14	4	9.74	0.41 (0.11–1.05)	− 2.65
Breast	74	74.25	1.00 (0.78–1.25)	− 0.08	–	–	–	–	70	73.82	0.95 (0.74–1.20)	− 1.77
Female genital system	31	28.75	1.08 (0.73–1.53)	0.69	–	–	–	–	31	28.75	1.08 (0.73–1.53)	1.04
Male genital system	54	50.95	1.06 (0.80–1.38)	0.93	54	50.95	1.06 (0.80–1.38)	2.75	–	–	–	–
Urinary bladder	20	19.1	1.05 (0.64–1.62)	0.28	14	12.84	1.09 (0.60–1.83)	1.04	6	6.25	0.90 (0.35–2.09)	− 0.12
Kidney and renal pelvis	21	14.39	1.46 (0.90–2.23)	2.02	13	7.38	1.76 (0.94–3.01)	5.08	8	7.01	1.14 (0.49–2.25)	0.46
Thyroid	26	7.78	3.34 (2.18–4.89)	5.58	7	1.58	4.42 (1.78–9.11)	4.9	19	6.2	3.06 (1.84–4.78)	5.92
Lymphoma	23	18.94	1.21 (0.77–1.82)	1.24	9	8.02	1.12 (0.51–2.13)	0.88	14	10.92	1.28 (0.70–2.15)	1.43
Leukemia	13	12.04	1.08 (0.57–1.85)	0.29	8	5.58	1.43 (0.62–2.82)	2.18	5	6.46	0.77 (0.25–1.81)	− 0.67

O, Observed; E, Expected; CI, confidence interval; EAR, excess absolute risk; SIR, standardized incidence ratio

#### Risk of SPMs as a function of time since G-NETs diagnosis

**Fig. 2:**
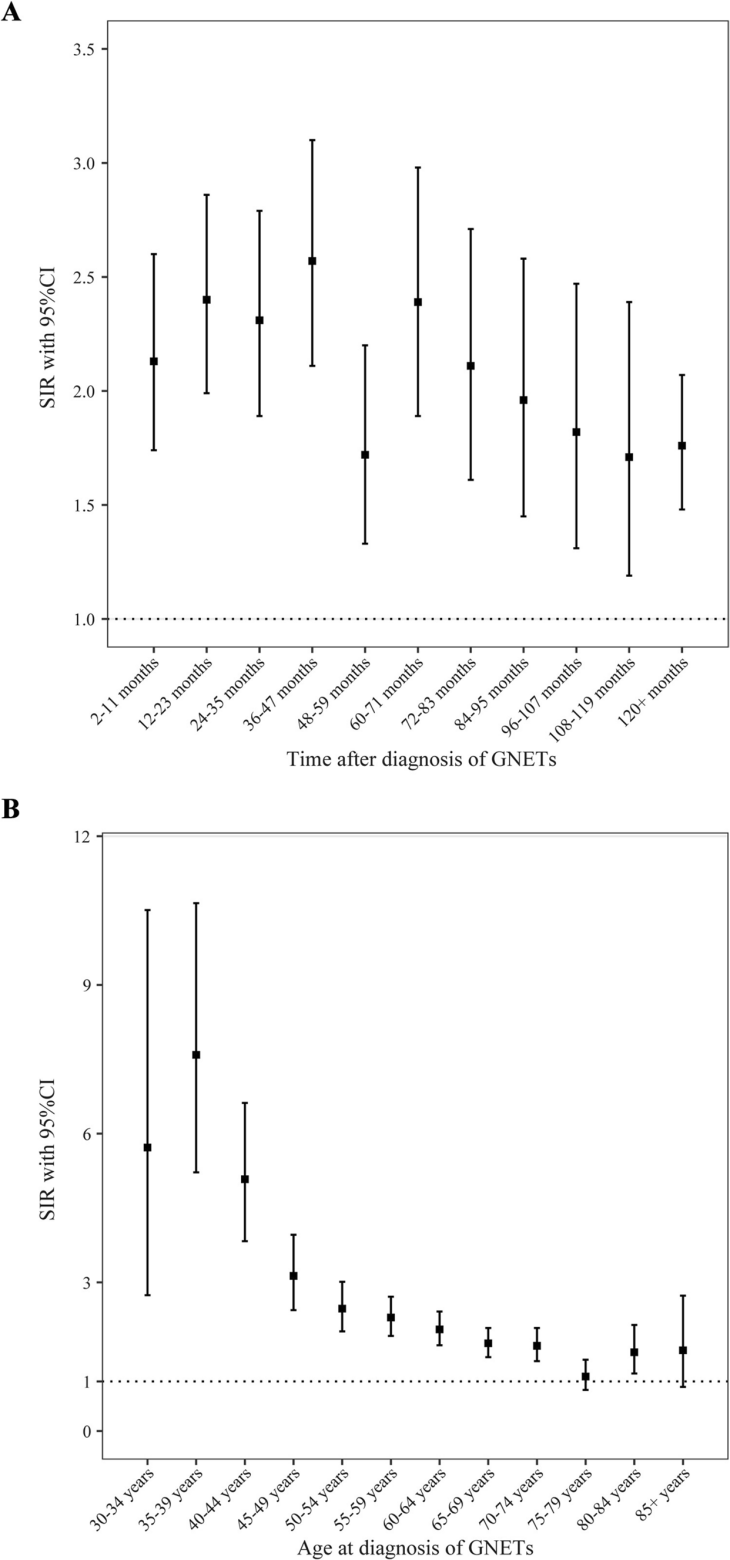
Stratified standardized incidence ratios for second primary malignancies of all cancers combined (**A**) by time after diagnosis of gastric neuroendocrine tumors (G-NETs) and **B** by age at diagnosis of G-NETs. SIR, standardized incidence ratio; CI, confidence interval

The risk of developing an SPM varied over time after the initial G-NET diagnosis. In the early post-diagnosis period (< 4 years), the risk was highest, with an SIR of 2.57, and gradually decreased over time, though it remained elevated even after 10 years (SIR 1.76; 95% CI 1.48–2.07) (Fig. 2A).

#### Risk of SPMs by gender

Female patients demonstrated a slightly higher SIR (2.18; 95% CI 2.00–2.36) compared to male patients (SIR 1.97; 95% CI 1.77–2.19), with males exhibiting a higher EAR (157.62 vs. 139.5 for females). Both genders showed increased risks for gastric cancer, small intestine cancer, hepatobiliary cancers, pancreatic cancer, and thyroid cancer. Additionally, an elevated risk of oral cavity and pharyngeal cancers was observed exclusively in male patients (Table 2).

#### Risk of SPMs by age at G-NET diagnosis

Patients diagnosed with G-NETs younger than 50 years exhibited markedly higher SIRs, with the highest observed in individuals aged 35–39 years (SIR 7.59; 95% CI 5.22–10.66). The risk decreased progressively with increasing age, and patients aged ≥ 75 years showed an SIR approaching 1.0 (Fig. 2B). Younger patients were also at higher risk for stomach cancer, with SIRs of 196.28 for those under 50 years, 59.63 for those aged 50–70 years, and 24.96 for those over 70 years (Table 3).

**Table 3:** Risk of specific second primary malignancies after first primary gastric neuroendocrine tumors by age at diagnosis in the USA from 2000 through 2021

	< 50 years				50–69 years				≥ 70 years			
	O	E	SIR (95% CI)	EAR	O	E	SIR (95% CI)	EAR	O	E	SIR (95% CI)	EAR
All sites	174	39.45	4.41 (3.78–5.12)	163.18	521	250.49	2.08 (1.91–2.27)	152.56	217	146.24	1.48 (1.29–1.70)	105.7
All solid tumors	169	35.54	4.76 (4.07–5.53)	161.86	477	220.96	2.16 (1.97–2.36)	144.4	202	122.15	1.65 (1.43–1.90)	119.27
Oral cavity and pharynx	0	1.05	0 (0.00–3.52)	− 1.27	12	5.89	2.04 (1.05–3.56)	3.45	2	2.77	0.72 (0.09–2.61)	− 1.15
Esophagus	1	0.25	3.93 (0.10–21.87)	0.9	6	2.34	2.56 (0.94–5.58)	2.06	3	1.4	2.14 (0.44–6.26)	2.39
Stomach	103	0.52	196.28 (160.21–238.05)	124.28	213	3.57	59.63 (51.89–68.19)	118.11	66	2.64	24.96 (19.31–31.76)	94.63
Small Intestine	2	0.23	8.82 (1.07–31.86)	2.15	14	1.37	10.18 (5.57–17.09)	7.12	3	0.74	4.07 (0.84–11.89)	3.38
Colorectal	4	3.63	1.10 (0.30–2.82)	0.45	14	20.89	0.67 (0.37–1.12)	− 3.89	14	15.58	0.90 (0.49–1.51)	− 2.36
Hepatobiliary	6	0.84	7.18 (2.63–15.62)	6.26	26	6.82	3.81 (2.49–5.59)	10.82	6	3.76	1.6 (0.59–3.47)	3.35
Pancreas	8	0.77	10.41 (4.50–20.52)	8.77	17	7.49	2.27 (1.32–3.64)	5.37	15	5.82	2.58 (1.44–4.25)	13.71
Lung and bronchus	4	2.76	1.45 (0.39–3.71)	1.5	30	33.49	0.90 (0.60–1.28)	− 1.97	29	23.18	1.25 (0.84–1.80)	8.69
Melanoma of the skin	2	2.22	0.90 (0.11–3.25)	− 0.27	5	11.11	0.45 (0.15–1.05)	− 3.44	6	6.67	0.90 (0.33–1.96)	− 1
Breast	13	10.35	1.26 (0.67–2.15)	3.21	46	44	1.05 (0.77–1.39)	1.13	15	19.9	0.75 (0.42–1.24)	− 7.32
Female genital system	7	3.81	1.84 (0.74–3.79)	3.87	16	17.57	0.91 (0.52–1.48)	− 0.89	8	7.36	1.09 (0.47–2.14)	0.95
Male genital system	4	3.32	1.21 (0.33–3.09)	0.83	34	34.74	0.98 (0.68–1.37)	− 0.41	16	12.9	1.24 (0.71–2.01)	4.63
Urinary bladder	2	0.74	2.72 (0.33–9.83)	1.53	13	9.61	1.35 (0.72–2.31)	1.91	5	8.75	0.57 (0.19–1.33)	− 5.6
Kidney and renal pelvis	4	1.47	2.72 (0.74–6.97)	3.07	7	8.74	0.80 (0.32–1.65)	− 0.98	10	4.18	2.39 (1.15–4.40)	8.69
Thyroid	9	2.06	4.37 (2.00–8.30)	8.42	15	4.53	3.31 (1.85–5.46)	5.91	2	1.2	1.67 (0.20–6.03)	1.2
Lymphoma	2	1.6	1.25 (0.15–4.51)	0.48	16	10.23	1.56 (0.89–2.54)	3.25	5	7.11	0.70 (0.23–1.64)	− 3.15
Leukemia	2	0.86	2.34 (0.28–8.44)	1.39	7	6.28	1.12 (0.45–2.30)	0.41	4	4.91	0.82 (0.22–2.09)	− 1.36

O, Observed; E, Expected; CI, confidence interval; EAR, excess absolute risk; SIR, standardized incidence ratio

#### Risk of SPMs by race

When analyzed by race, patients categorized as "Other" exhibited the highest SIR for SPMs (2.60; 95% CI 2.01–3.32), followed by Black patients (SIR 2.07; 95% CI 1.70–2.49) and White patients (SIR 2.06; 95% CI 1.91–2.21). White patients had the highest risk of stomach cancer (SIR 63.88; 95% CI 57.00–71.38), compared to 45.91 (95% CI 31.19–65.16) in the other group and 32.60 (95% CI 23.07–44.75) in Black patients (Table 4).

**Table 4:** Risk of specific second primary malignancies after first primary gastric neuroendocrine tumors by race in the USA from 2000 through 2021

	White				Black				Others			
	O	E	SIR (95% CI)	EAR	O	E	SIR (95% CI)	EAR	O	E	SIR (95% CI)	EAR
All sites	736	357.46	2.06 (1.91–2.21)	147.8	111	53.75	2.07 (1.70–2.49)	130.61	65	24.96	2.60 (2.01–3.32)	149.56
All solid tumors	685	309.46	2.21 (2.05–2.39)	146.63	101	47.33	2.13 (1.74–2.59)	122.43	62	21.85	2.84 (2.18–3.64)	149.95
Oral cavity and pharynx	10	8.21	1.22 (0.58–2.24)	0.7	1	0.91	1.10 (0.03–6.15)	0.21	3	0.59	5.05 (1.04–14.76)	8.99
Esophagus	7	3.34	2.10 (0.84–4.32)	1.43	2	0.45	4.43 (0.54–15.99)	3.53	1	0.21	4.86 (0.12–27.10)	2.97
Stomach	313	4.9	63.88 (57.00–71.36)	120.3	38	1.17	32.60 (23.07–44.75)	84.03	31	0.68	45.91 (31.19–65.16)	113.26
Small intestine	15	1.76	8.52 (4.77–14.06)	5.17	1	0.46	2.16 (0.05–12.02)	1.22	3	0.12	26.02 (5.37–76.03)	10.77
Colorectal	27	31.9	0.85 (0.56–1.23)	− 1.91	3	5.62	0.53 (0.11–1.56)	− 5.98	2	2.58	0.77 (0.09–2.80)	− 2.17
Hepatobiliary	26	8.67	3.00 (1.96–4.39)	6.77	6	1.57	3.82 (1.40–8.31)	10.1	6	1.18	5.10 (1.87–11.10)	18.02
Pancreas	32	11.2	2.86 (1.95–4.03)	8.12	7	1.98	3.54 (1.42–7.29)	11.45	1	0.89	1.12 (0.03–6.23)	0.4
Lung and bronchus	48	48.85	0.98 (0.72–1.30)	− 0.33	11	7.31	1.51 (0.75–2.69)	8.43	4	3.28	1.22 (0.33–3.13)	2.7
Melanoma of the skin	13	19.33	0.67 (0.36–1.15)	− 2.47	0	0.11	0.00 (0.00–33.91)	− 0.25	0	0.56	0 (0.00–6.57)	− 2.1
Breast	62	60.17	1.03 (0.79–1.32)	0.71	9	9.76	0.92 (0.42–1.75)	− 1.74	3	4.31	0.70 (0.14–2.03)	− 4.91
Female genital system	23	23.13	0.99 (0.63–1.49)	− 0.05	6	3.95	1.52 (0.56–3.31)	4.68	2	1.67	1.20 (0.15–4.34)	1.25
Male genital system	44	39.88	1.10 (0.80–1.48)	1.61	8	8.36	0.96 (0.41–1.89)	− 0.82	2	2.72	0.74 (0.09–2.66)	− 2.67
Urinary bladder	17	16.93	1.00 (0.58–1.61)	0.03	2	1.32	1.52 (0.18–5.49)	1.56	1	0.85	1.17 (0.03–6.54)	0.55
Kidney and renal pelvis	18	11.64	1.55 (0.92–2.44)	2.48	1	1.97	0.51 (0.01–2.83)	− 2.21	2	0.78	2.57 (0.31–9.29)	4.56
Thyroid	21	6.44	3.26 (2.02–4.98)	5.68	4	0.75	5.33 (1.45–13.66)	7.41	1	0.59	1.69 (0.04–9.41)	1.52
Lymphoma	20	16.25	1.23 (0.75–1.90)	1.46	3	1.56	1.92 (0.40–5.60)	3.27	0	1.13	0 (0.00–3.27)	− 4.21
Leukemia	11	10.34	1.06 (0.53–1.90)	0.26	1	1.11	0.90 (0.02–5.02)	− 0.25	1	0.59	1.70 (0.04–9.47)	1.54

O, Observed; E, Expected; CI, confidence interval; EAR, excess absolute risk; SIR, standardized incidence ratio

#### Risk of SPMs by marital status and surgery

Married patients exhibited a slightly higher SIR (2.15; 95% CI 1.97–2.34) compared to unmarried patients (SIR 2.01; 95% CI 1.82–2.23), with a higher EAR (158.1 vs. 130.83). Regarding surgical treatment, patients who underwent surgery for their initial G-NET had a slightly lower SIR (2.06; 95% CI 1.90–2.24) compared to those who did not undergo surgery (SIR 2.14; 95% CI 1.92–2.38) (Supplementary Tables 1 and 2).

## Discussion

This large-scale cohort study examines the relationship between the initial primary malignancy and the subsequent development of SPMs among survivors of G-NETs in the United States from 2000 to 2021. To our knowledge, this is the first systematic investigation of the risk of SPMs in G-NET survivors. The cohort comprised 5,072 G-NET patients, of whom 912 (18.0%) were diagnosed with an SPM during the follow-up period. The median interval between the diagnosis of the first and second malignancy was 34.3 months. Overall, survivors of G-NETs demonstrated a 109% higher risk of developing SPMs relative to the general U.S. population, with an EAR of 145.64 cases per 10,000 person-years. The most commonly observed SPMs included cancers of the stomach, small intestine, thyroid, and hepatobiliary system. Our results can guide clinicians in identifying high-risk groups among G-NET survivors who may benefit from more intensive monitoring and early intervention.

A study conducted at a tertiary referral center in the Netherlands investigated the incidence of SPMs in patients diagnosed with gastroenteropancreatic neuroendocrine tumors (GEP-NETs). The cohort included 459 patients who were diagnosed between 2000 and 2009, with 67 individuals (13.7%) subsequently diagnosed with a SPM. The findings indicated that synchronous SPMs—defined as those occurring within 6 months before or after the GEP-NET diagnosis—were significantly more prevalent in this population, particularly colorectal cancers. Despite this, the authors concluded that routine screening for SPMs in GEP-NET patients is not justified, as the elevated risk was confined to synchronous malignancies, rather than a broader increased risk for subsequent cancers [18]. In contrast, a more extensive, population-based analysis utilizing data from the SEER registries assessed the risk of SPMs in NET patients from 2000 to 2016. Among 58,596 patients, 4612 (7.9%) developed a SPM. This study identified a statistically significant increase in the risk of SPMs in NET patients, with an SIR of 1.35 (95% CI 1.31–1.39). The cumulative five-year incidence of SPMs varied by the primary site of the NET, ranging from 3.8% for pancreatic NETs to 5.9% for gastrointestinal NETs [17]. Further investigations focusing on patients with pancreatic and gastrointestinal NETs corroborated these findings, revealing that individuals with these tumor types are at a considerably higher risk of developing additional malignancies [19].

Our analysis of SPMs in relation to key patient characteristics provides valuable insights into risk stratification and the optimization of surveillance protocols. The risk of developing SPMs decreased progressively with increasing age, which suggests a potential age-dependent protective effect against the development of additional malignancies. This trend could be attributed to several underlying factors. Firstly, the shorter life expectancy typical of older individuals may reduce the likelihood of developing a second malignancy within the time frame preceding death [20]. Additionally, age-related immune system changes, such as immunosenescence, could impair the body’s capacity to detect and eliminate malignant cells, thereby attenuating cancer progression and reducing the occurrence of SPMs [21, 22]. Moreover, older patients are less likely to undergo routine cancer screenings, which may further contribute to the observed decline in SPM incidence with increasing age. For instance, a study conducted in Taiwan involving 1350 newly diagnosed NET patients revealed an increased risk of second malignancies, particularly among individuals aged 70 years or older [23]. In clinical practice, the management of older patients with G-NETs requires a nuanced approach. Clinicians must balance the potential benefits of intensive SPM surveillance with the limitations imposed by reduced life expectancy. Such considerations are essential for developing personalized follow-up strategies that integrate both chronological age and overall health status, thereby optimizing patient care and ensuring resource-efficient monitoring. It is also important to consider that the SEER database has a higher proportion of older individuals. This demographic characteristic of the database could potentially influence the observed decrease in SPM risk with advancing age. Specifically, the lower representation of younger patients in the SEER data might limit our ability to fully characterize the risk of SPMs in this population.

As time progresses, the risk of developing a SPM decreases; however, even after 10 years, the risk remains elevated, indicating that the initial diagnosis of G-NETs and the long-term effects of the disease may continue to predispose patients to subsequent malignancies. One potential explanation for this sustained risk is that individuals with a history of G-NET may experience persistent immunological alterations or chronic inflammation, which could contribute to a heightened susceptibility to new malignancies [24, 25]. Additionally, the prolonged elevated risk may reflect underlying genetic predispositions or pre-existing conditions that increase the likelihood of developing multiple cancers. For instance, patients with inherited cancer syndromes or a family history of malignancy may be more prone to the development of secondary malignancies [26, 27]. Furthermore, the genetic mutations or tumor suppressor gene alterations that initially drove G-NET tumorigenesis may also play a role in the pathogenesis of secondary cancers, suggesting a shared molecular pathway between primary and secondary malignancies. These findings highlight the need for ongoing surveillance in patients with G-NETs, as the elevated risk of secondary malignancies persists beyond 10 years and may remain a significant concern throughout the patient’s lifetime.

Our study indicates that while women may exhibit a higher SIR for SPMs, the EAR is greater in men. This disparity may be attributed to gender differences in lifestyle factors that predispose individuals to certain cancers. Men are more likely to engage in behaviors such as smoking, alcohol consumption, and obesity, all of which are known to increase the risk of various malignancies [28, 29]. These lifestyle factors may partly explain the higher EAR observed in males. Smoking, for example, is a well-established risk factor for numerous cancers, including those of the oral cavity, pharynx, and lungs. The higher prevalence of smoking among men may account for the elevated risk of oral and pharyngeal cancers observed exclusively in male patients. Similarly, alcohol consumption is a known contributor to the development of liver cancer, which may further elevate the risk in males. Both sexes demonstrated increased risks for malignancies of the gastric, small intestine, hepatobiliary system, pancreas, and thyroid, suggesting that the biological mechanisms underlying G-NETs might predispose patients to these specific malignancies. These malignancies, often associated with the gastrointestinal and endocrine systems, may share common pathways of tumorigenesis with G-NETs. For instance, neuroendocrine tumors are known to secrete bioactive amines that can influence cellular function and growth in other tissues, potentially heightening susceptibility to secondary malignancies [30, 31].

Racial disparities were detected in our study. White patients were found to have a risk comparable to that of Black patients, suggesting that while genetic factors may contribute, variations in the SIRs for SPMs across racial groups could also be influenced by disparities in healthcare access and treatment. Notably, White patients exhibited the highest risk for stomach cancer compared to other racial groups. This disparity may be attributed to specific lifestyle factors, such as dietary habits, smoking prevalence, and alcohol consumption, which have been linked to higher incidences of stomach cancer in certain populations.

This cohort study offers several notable strengths, including a large sample size, an extended follow-up period, and a population-based design, all of which enhance the external validity of the results, allowing for generalization to the wider U.S. population. Nonetheless, certain limitations must be acknowledged. First, while the SEER database provides robust population-level data, it lacks granular details on socioeconomic status, environmental exposures (e.g., smoking, alcohol use, dietary habits), and genetic predispositions (e.g., hereditary syndromes like MEN1), which may confound the observed risks of SPMs. Second, treatment specifics, such as chemotherapy regimens, radiation doses, or prolonged use of somatostatin analogs, were unavailable, limiting our ability to assess their potential role in SPM development. Third, while the substantial sample size and extended follow-up duration provide valuable insights, the relatively low frequency of the events under study resulted in wide confidence intervals. Finally, the assumption of a two-month latency period between cancer diagnoses may have contributed to an overestimation of the SPM incidence. Therefore, caution is warranted in the interpretation of these findings.

Over the next 5 years, we anticipate transformative shifts in this field. Personalized risk prediction models incorporating genetic, clinical, and lifestyle data will likely become standard tools for guiding SPM surveillance. Advances in liquid biopsy technologies may enable early detection of secondary malignancies through circulating tumor DNA or neuroendocrine-specific biomarkers. Additionally, immunotherapy trials targeting immune checkpoints (e.g., PD-L1 inhibitors) in G-NETs could reveal whether these therapies mitigate SPM risk by modulating the tumor microenvironment.

## Conclusion

This study highlights the risk of SPMs in patients with G-NETs, which remains elevated even after 10 years. Gender, age, and racial disparities significantly influence SPM risk, particularly for cancer of gastrointestinal and endocrine systems. These findings emphasize the need for personalized surveillance strategies that consider these factors. Clinicians should integrate these variables into follow-up care plans. Further research is needed to explore underlying mechanisms and develop targeted prevention strategies to reduce SPM incidence and improve outcomes.

## Supplementary Information

Below is the link to the electronic supplementary material.

Supplementary file1 (DOCX 21 KB)

Supplementary file2 (DOCX 21 KB)

## Data Availability

All data used in this study can be acquired from the SEER*Stat software (version 8.3.9).
