# Supplementary material for: The persistent risk of secondary malignancies in gastric neuroendocrine tumor survivors: a population-based analysis
Source: Clin Exp Med. 2025 May 14;25(1):158. doi: 10.1007/s10238-025-01706-y (PMC12078349; doi:10.1007/s10238-025-01706-y)
Supplement: Supplementary file 1 — Supplementary file1 (DOCX 21 KB) [file 10238_2025_1706_MOESM1_ESM.docx]

| **Supplementary table 1.** Risk of specific second primary malignancies after first primary gastric neuroendocrine tumors by marital status in the USA from 2000 through 2021. | | | | | | | | |
| --- | --- | --- | --- | --- | --- | --- | --- | --- |
|  | **Married** | | | | **Unmarried** | | | |
|  | O | E | SIR (95% CI) | EAR | O | E | SIR (95% CI) | EAR |
| All Sites | 524 | 243.55 | 2.15 (1.97-2.34) | 158.1 | 388 | 192.62 | 2.01 (1.82-2.23) | 130.83 |
| All Solid Tumors | 488 | 211.64 | 2.31 (2.11-2.52) | 155.79 | 360 | 167.01 | 2.16 (1.94-2.39) | 129.24 |
| Oral Cavity and Pharynx | 10 | 5.77 | 1.73 (0.83-3.19) | 2.39 | 4 | 3.94 | 1.02 (0.28-2.60) | 0.04 |
| Esophagus | 3 | 2.43 | 1.23 (0.25-3.61) | 0.32 | 7 | 1.57 | 4.47 (1.80-9.21) | 3.64 |
| Stomach | 216 | 3.77 | 57.37 (49.97-65.55) | 119.64 | 166 | 2.98 | 55.79 (47.62-64.95) | 109.17 |
| Small Intestine | 15 | 1.27 | 11.83 (6.62-19.51) | 7.74 | 4 | 1.07 | 3.74 (1.02-9.57) | 1.96 |
| Colorectal | 13 | 21.73 | 0.60 (0.32-1.02) | -4.92 | 19 | 18.37 | 1.03 (0.62-1.62) | 0.42 |
| Hepatobiliary | 22 | 6.55 | 3.36 (2.11-5.09) | 8.71 | 16 | 4.87 | 3.29 (1.88-5.34) | 7.45 |
| Pancreas | 23 | 7.57 | 3.04 (1.93-4.56) | 8.7 | 17 | 6.51 | 2.61 (1.52-4.18) | 7.03 |
| Lung and Bronchus | 30 | 32.96 | 0.91 (0.61-1.30) | -1.67 | 33 | 26.48 | 1.25 (0.86-1.75) | 4.37 |
| Melanoma of the Skin | 9 | 12.01 | 0.75 (0.34-1.42) | -1.7 | 4 | 7.98 | 0.50 (0.14-1.28) | -2.67 |
| Breast | 45 | 36.04 | 1.25 (0.91-1.67) | 5.05 | 29 | 38.2 | 0.76 (0.51-1.09) | -6.16 |
| Female Genital System | 18 | 13.93 | 1.29 (0.77-2.04) | 2.29 | 13 | 14.81 | 0.88 (0.47-1.50) | -1.21 |
| Male Genital System | 37 | 34.34 | 1.08 (0.76-1.48) | 1.5 | 17 | 16.61 | 1.02 (0.60-1.64) | 0.26 |
| Urinary Bladder | 12 | 11.79 | 1.02 (0.53-1.78) | 0.12 | 8 | 7.31 | 1.09 (0.47-2.16) | 0.46 |
| Kidney and Renal Pelvis | 12 | 8.28 | 1.45 (0.75-2.53) | 2.1 | 9 | 6.1 | 1.47 (0.67-2.80) | 1.94 |
| Thyroid | 14 | 4.22 | 3.32 (1.81-5.57) | 5.51 | 12 | 3.56 | 3.37 (1.74-5.88) | 5.65 |
| Lymphoma | 16 | 10.66 | 1.50 (0.86-2.44) | 3.01 | 7 | 8.29 | 0.84 (0.34-1.74) | -0.86 |
| Leukemia | 5 | 6.81 | 0.73 (0.24-1.71) | -1.02 | 8 | 5.23 | 1.53 (0.66-3.02) | 1.86 |

O, Observed; E, Expected; CI, confidence interval; EAR, excess absolute risk; SIR, standardized incidence ratio.
