# Supplementary material for: The persistent risk of secondary malignancies in gastric neuroendocrine tumor survivors: a population-based analysis
Source: Clin Exp Med. 2025 May 14;25(1):158. doi: 10.1007/s10238-025-01706-y (PMC12078349; doi:10.1007/s10238-025-01706-y)
Supplement: Supplementary file 2 — Supplementary file2 (DOCX 21 KB) [file 10238_2025_1706_MOESM2_ESM.docx]

| Supplementary table 2. Risk of specific second primary malignancies after first primary gastric neuroendocrine tumors by surgery in the USA from 2000 through 2021. | | | | | | | | |
| --- | --- | --- | --- | --- | --- | --- | --- | --- |
|  | **Surgery** | | | | **No/unknown surgery** | | | |
|  | O | E | SIR (95% CI) | EAR | O | E | SIR (95% CI) | EAR |
| All Sites | 565 | 273.88 | 2.06 (1.90-2.24) | 138.29 | 347 | 162.29 | 2.14 (1.92-2.38) | 158.95 |
| All Solid Tumors | 525 | 238.22 | 2.20 (2.02-2.40) | 136.22 | 323 | 140.43 | 2.30 (2.06-2.57) | 157.11 |
| Oral Cavity and Pharynx | 9 | 6.19 | 1.45 (0.67-2.76) | 1.34 | 5 | 3.52 | 1.42 (0.46-3.32) | 1.27 |
| Esophagus | 8 | 2.53 | 3.16 (1.36-6.22) | 2.6 | 2 | 1.46 | 1.37 (0.17-4.94) | 0.46 |
| Stomach | 240 | 4.21 | 57.03 (50.04-64.72) | 112.01 | 142 | 2.53 | 56.07 (47.23-66.09) | 120.02 |
| Small Intestine | 14 | 1.47 | 9.50 (5.19-15.94) | 5.95 | 5 | 0.87 | 5.78 (1.88-13.49) | 3.56 |
| Colorectal | 21 | 24.85 | 0.85 (0.52-1.29) | -1.83 | 11 | 15.25 | 0.72 (0.36-1.29) | -3.66 |
| Hepatobiliary | 22 | 7.2 | 3.05 (1.91-4.63) | 7.03 | 16 | 4.21 | 3.80 (2.17-6.16) | 10.14 |
| Pancreas | 22 | 8.69 | 2.53 (1.59-3.83) | 6.32 | 18 | 5.39 | 3.34 (1.98-5.28) | 10.85 |
| Lung and Bronchus | 36 | 36.97 | 0.97 (0.68-1.35) | -0.46 | 27 | 22.47 | 1.20 (0.79-1.75) | 3.9 |
| Melanoma of the Skin | 8 | 12.67 | 0.63 (0.27-1.24) | -2.22 | 5 | 7.33 | 0.68 (0.22-1.59) | -2 |
| Breast | 47 | 46.04 | 1.02 (0.75-1.36) | 0.46 | 27 | 28.2 | 0.96 (0.63-1.39) | -1.04 |
| Female Genital System | 16 | 17.81 | 0.90 (0.51-1.46) | -0.86 | 15 | 10.93 | 1.37 (0.77-2.26) | 3.5 |
| Male Genital System | 36 | 33.59 | 1.07 (0.75-1.48) | 1.15 | 18 | 17.37 | 1.04 (0.61-1.64) | 0.55 |
| Urinary Bladder | 9 | 11.98 | 0.75 (0.34-1.43) | -1.41 | 11 | 7.12 | 1.54 (0.77-2.76) | 3.34 |
| Kidney and Renal Pelvis | 12 | 9.14 | 1.31 (0.68-2.29) | 1.36 | 9 | 5.24 | 1.72 (0.79-3.26) | 3.23 |
| Thyroid | 16 | 5.02 | 3.19 (1.82-5.17) | 5.21 | 10 | 2.76 | 3.62 (1.74-6.66) | 6.23 |
| Lymphoma | 12 | 11.82 | 1.01 (0.52-1.77) | 0.08 | 11 | 7.12 | 1.55 (0.77-2.77) | 3.34 |
| Leukemia | 9 | 7.5 | 1.20 (0.55-2.28) | 0.71 | 4 | 4.54 | 0.88 (0.24-2.25) | -0.47 |

O, Observed; E, Expected; CI, confidence interval; EAR, excess absolute risk; SIR, standardized incidence ratio.
